# Cherry Juice Improves Memory and Anxiety by Modulating Cell Number in the Hippocampus of Male Rat Pups Infected with Lipopolysaccharides During Gestation and Gestation-Lactation

**DOI:** 10.3390/ijms26125642

**Published:** 2025-06-12

**Authors:** Juan J. Virgen-Gen, Mayvi Alvarado-Olivarez, Rosa I. Guzmán-Gerónimo, Paola F. González-Nieto, Ana G. Gutiérrez-García, Rosa M. Oliart-Ros

**Affiliations:** 1 Instituto de Neuroetología, Universidad Veracruzana, Xalapa 91190, Veracruz, Mexico; juanjesusvgen@outlook.com (J.J.V.-G.); pglez5@hotmail.com (P.F.G.-N.); angutierrez@uv.mx (A.G.G.-G.); 2 Instituto de Ciencias Básicas, Universidad Veracruzana, Xalapa 91190, Veracruz, Mexico; 3 Unidad de Investigación y Desarrollo en Alimentos (UNIDA), Tecnológico Nacional de México, Formando Veracruz 91897, Veracruz, Mexico; rosa.or@veracruz.tecnm.mx

**Keywords:** cherry juice, neuroprotection, leukocyte, anxiogenic, locomotor

## Abstract

Exposure to lipopolysaccharides (LPS) during pregnancy have been linked to alterations in the offspring’s central nervous system. Cherries are a source of anthocyanins, which possess neuroprotective properties. The study aimed to evaluate the neuroprotective effects of cherry juice (CJ) on memory and hippocampal cell counts in offspring exposed to LPS during gestation and the gestation-lactation periods. At postnatal day 90, rat pups were divided into five groups: Control (saline solution), LPS-G (LPS during gestation), LPS-G+CJ (LPS during gestation+CJ), LPS-GL (LPS during gestation-lactation), and LPS-GL+CJ (LPS during gestation-lactation+CJ). A battery of behavioral tests was conducted to assess short- and long-term memory and anxiety-like behavior. Histological analysis was performed on hippocampal regions. Leukocyte levels were measured as markers of systemic inflammation. Results showed that pups in the LPS-G and LPS-GL groups exhibited impaired memory, increased anxiety-like behavior, elevated leukocyte levels, and reduced cell counts in the dentate gyrus and CA1 regions, as well as in CA2 (LPS-G) and CA3 (LPS-GL). Cherry juice administration in the LPS-G+CJ and LPS-GL+CJ groups improved memory performance, normalized leukocyte levels, and restored hippocampal cell counts. These findings suggest that cherry juice exerts neuroprotective effects against LPS-induced neuroinflammation during gestation and the gestation-lactation periods.

## 1. Introduction

Lipopolysaccharides (LPS), commonly referred to as endotoxins, constitute the principal component of Gram-negative (G [-]) bacteria and are in the outer membrane of their cell wall [1]. These compounds play a crucial role in the pathogenesis of the inflammatory system due to their high infectivity and survival capabilities when colonizing the host, thereby activating various immune response mechanisms [2,3]. Studies previously indicate that LPS can affect the levels of leukocytes in the blood, which are important cells in the inflammatory response [4].

Maternal infection by LPS stimulates the immune system, increasing leukocyte counts in pregnant rats, also affected organs such as the brain [5,6,7,8], thereby disrupting neurogenesis in offspring, heightening the risk of neurodegenerative diseases in adulthood. Maternal nutrition is a key factor in the development of the brain in the offspring. In recent years, biological compounds such as polyphenols including anthocyanins have emerged as potential alternatives to prevent and treatment of neurodegenerative diseases [9,10,11,12]. These compounds possess multifunctional properties such as antioxidant, anti-inflammatory, and neuroprotector activity [13,14]. Previous studies reported that administration of a polyphenol concentrate normalized the quantity and functional activity of peripheral blood neutrophils and peritoneal macrophages, also decreased the number of lymphocytes in an in vivo model of diabetes [15]. In addition, polyphenols could reduce the brain injury in the fetus, newborn, and offspring [16,17]. Also in vitro studies have reported that polyphenols ameliorate LPS-induced memory impairment [18] and reduction in anxiety-like and depression-like behaviors in rats [19].

Consumption of phenolic-rich foods, such as red fruits has been associated with beneficial effects in memory and learning in both animals and humans [20,21]. Among these fruits, studies have reported that blueberries, cranberries, blackberries, and strawberries contributed to improvements in cognitive functions, particularly in learning and memory [22]. A recent clinical study showed that tart cherry consumption improves sustained attention, feelings of alertness and mental fatigue in middle-aged adults [23]. In addition, a study by our group using an in vitro model of acute gout attack demonstrated antioxidant and anti-inflammatory effects of cherry extract [24]. Recently, it has been reported that foods rich in anthocyanins such as blackberry juice and blue corn tortilla have been linked to enhanced neuronal proliferation in the offspring during embryonic neurogenesis [17,25]. Therefore, the aim of this study was to evaluate short- and long-term memory, anxiety and the number of cells in hippocampal regions in offspring exposed to maternal infection by LPS during the gestational (G) and gestational-lactational (GL) periods, as well as the biological effects of cherry juice in the offspring exposed to maternal infection by LPS at postnatal day (P) 90.

## 2. Results

### 2.1. Effects of Cherry Juice in Maternal Infection by LPS on Behavioral Response

**Figure 1:**
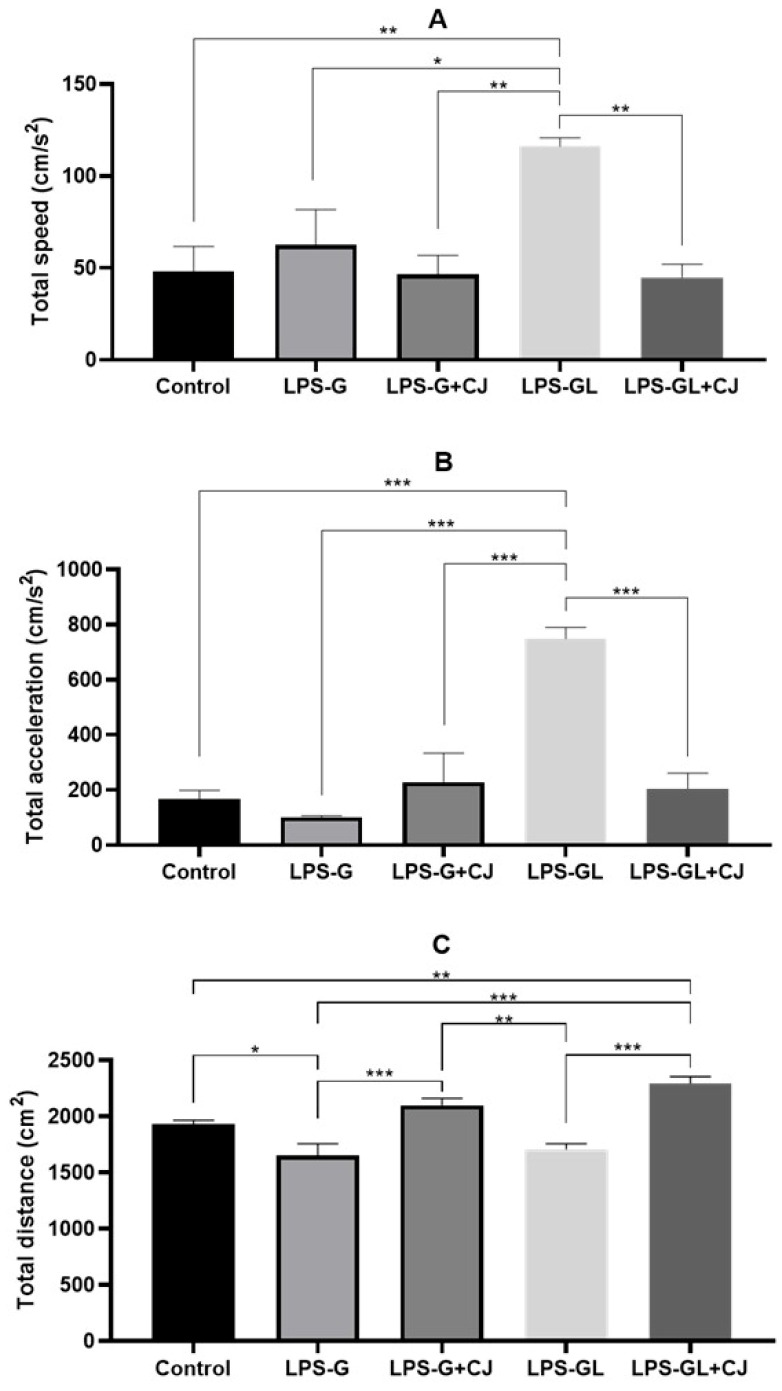
Mean ± SEM. Effects of cherry juice in maternal infection by lipopolysaccharide on the behavioral response of adult P90 rats in the open field (OP) test. (**A**) Total speed; (**B**) Total acceleration; (**C**) Total distance. * *p* < 0.01, ** *p* < 0.001, *** *p* < 0.0001. (Tukey’s post hoc). n = 6. LPS-G: LPS during gestation, LPS-G+CJ: LPS during gestation + cherry juice, LPS-GL: LPS during gestation-lactation, LPS-GL+CJ: LPS during gestation-lactation + cherry juice.

During the OF test, the offspring corresponding to the LPS-GL group showed significant differences with respect to the control and LPS-GL+CJ groups in total speed (F_4,25_ = 6.22, *p* = 0.0013) and total acceleration (F_4,25_ = 20.17, *p* < 0.0001) (Figure 1A,B). In addition, the offspring from the LPS-G group showed a decrease in total distance compared to the control group (F_4,25_ = 16.45, *p* < 0.0001). However, the administration of cherry juice in the LPS-G+CJ and LPS-GL-CJ groups increased the total distance (*p* < 0.05); it was statistically equal to the control group compared to the LPS exposed groups (Figure 1C).

**Figure 2:**
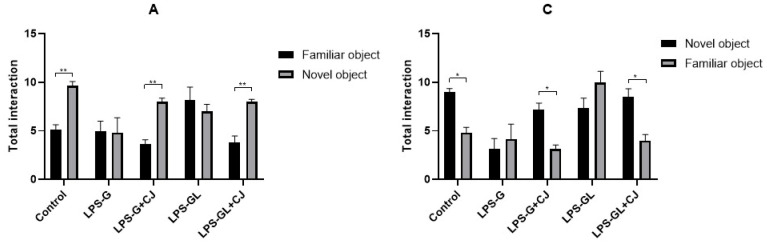

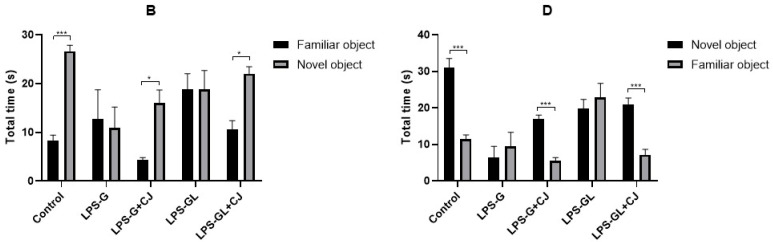
Mean ± SEM. Effects of cherry juice in maternal infection by lipopolysaccharide on the behavioral response of adult P90 rats in the novel object recognition (NOR) test. (**A**) Total numbers of interactions in short-term memory (STM); (**B**) Total time of interaction in short-term memory (STM); (**C**) Total numbers of interactions in long-term memory (LTM); (**D**) total time of interaction in long-term memory (LTM). * *p* < 0.01, ** *p* < 0.001, *** *p* < 0.0001 (Tukey’s post hoc). n = 6. LPS-G: LPS during gestation, LPS-G+CJ: LPS during gestation + cherry juice, LPS-GL: LPS during gestation-lactation, LPS-GL+CJ: LPS during gestation-lactation + cherry juice.

**Figure 3:**
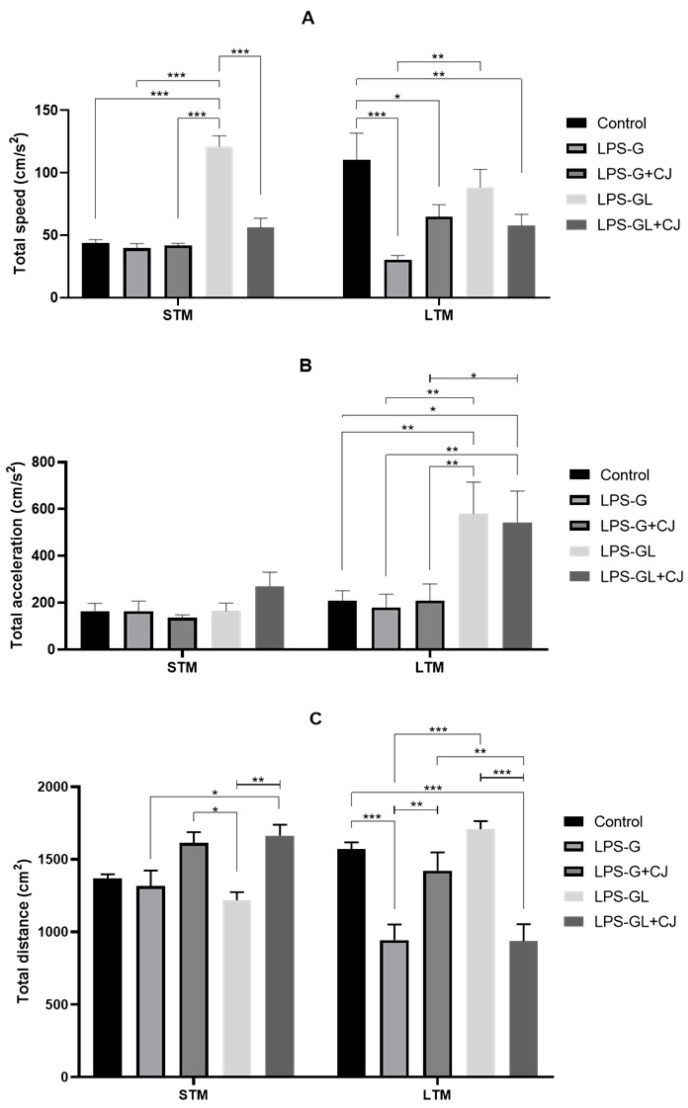
Mean ± SEM. Effects of cherry juice in maternal infection by lipopolysaccharide on the behavioral response of adult P90 rats in the novel object recognition (NOR) test. (**A**) Total speed; (**B**) Total acceleration; (**C**) Total distance. * *p* < 0.01, ** *p* < 0.001, *** *p* < 0.0001 (Tukey’s post hoc). n = 6. LPS-G: LPS during gestation, LPS-G+CJ: LPS during gestation + cherry juice, LPS-GL: LPS during gestation-lactation, LPS-GL+CJ: LPS during gestation-lactation + cherry juice.

During the NOR test, the LPS-G and LPS-GL groups interacted and spent more time with familiar objects during the STM (F_4,25_ = 6.84, *p* = 0.0007) and LTM (F_4,25_ = 6.01, *p* = 0.0016) phases, whereas the LPS-G+CJ and LPS-GL+CJ groups interacted and spent more time with novel objects, being statistically equal to the control group during the STM (F_4,25_ = 6.21, *p* = 0.0013) and LTM (F_4,25_ = 19.26, *p* < 0.0001) phases (Figure 2). In addition, during the STM phase, the LPS-GL group showed an increase in total speed compared to the experimental groups (F_4,50_ = 7.00, *p* = 0.0001). However, the LPS-G+CJ and LPS-GL+CJ groups were statistically equal to the control group (*p* < 0.05). On the other hand, during the LTM phase, all experimental groups showed lower values for total speed than the control group (F_4.50_ = 7.99, *p* < 0.0001) (Figure 3A). Moreover, during the LTM phase, the LPS-GL and LPS-GL+CJ groups showed an increment in total acceleration compared to the control group (F_4,50_ = 2.74, *p* = 0.0387) (Figure 3B). With respect to the total distance, the offspring of the LPS-GL+CJ group showed an increase compared to the LPS-GL group during the STM phase (F_4,50_ = 5.22, *p* = 0.0048). However, the LPS-GL+CJ group showed a decrease in total distance compared to the LPS-GL group during the LTM phase (F_4,50_ = 9.05, *p* < 0.0001). In addition, the LPS-G and LPS-GL groups decreased the total distance compared to the control group (F_4,50_ = 15.68, *p* < 0.0001) (Figure 3C).

**Figure 4:**
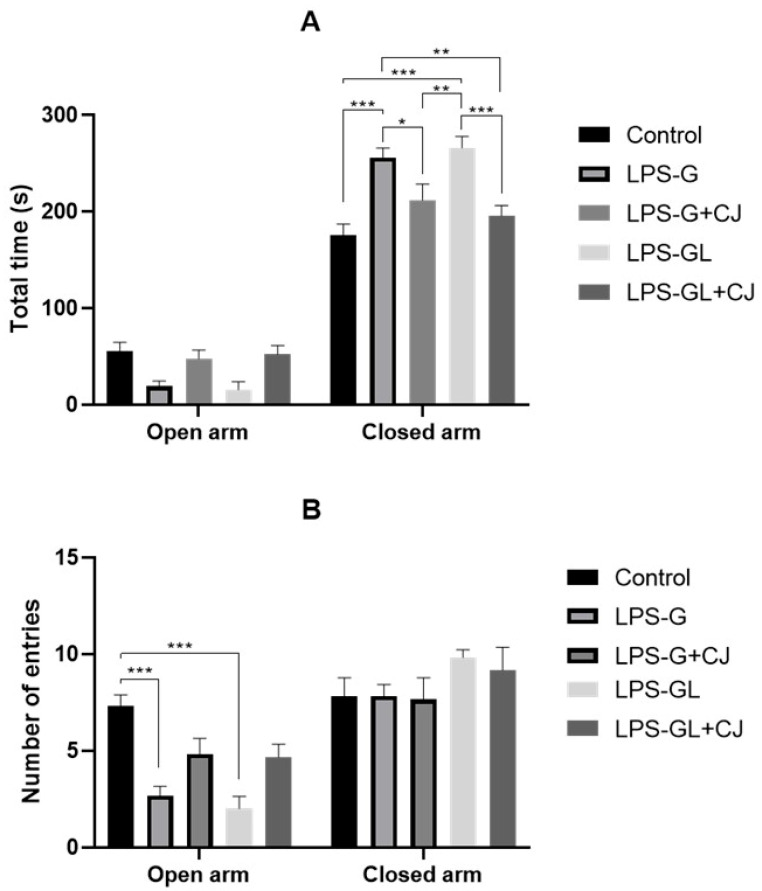
Mean ± SEM. Effects of cherry juice in maternal infection by lipopolysaccharide on the behavioral response of adult P90 rats in the elevated plus maze (EPM) test. (**A**) Total time spent in open and closed arms; (**B**) Number of entries into open and closed arms. * *p* < 0.01, ** *p* < 0.001, *** *p* < 0.0001 (Tukey’s post hoc). n = 6. LPS-G: LPS during gestation, LPS-G+CJ: LPS during gestation + cherry juice, LPS-GL: LPS during gestation-lactation, LPS-GL+CJ: LPS during gestation-lactation + cherry juice.

For the EPM test, no significant differences were observed in the time spent in open arms for any of the experimental groups. However, the LPS-G and LPS-GL groups were statistically different from the control, LPS-G+CJ and LPS-GL+CJ groups in the time spent in the closed arms (F_4,50_ = 15.13, *p* < 0.0001) (Figure 4A). In addition, the LPS-G and LPS-GL showed significant differences in the number of entries to the open arm compared to the control group (F_4,50_ = 6.08, *p* = 0.0005) (Figure 4B).

### 2.2. Leukocyte Analysis

**Figure 5:**
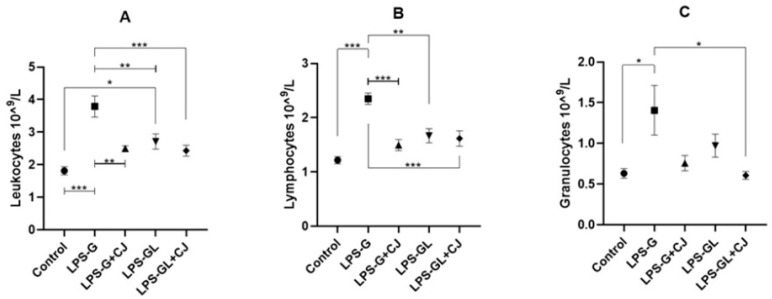
Mean ± SEM. Effects of cherry juice in maternal infection by lipopolysaccharide on immune system in blood. (**A**) Total leukocytes count; (**B**) Total lymphocytes count; (**C**) Total granulocytes count. * *p* < 0.01, ** *p* < 0.001, *** *p* < 0.0001. (Tukey’s post hoc). n = 6. LPS-G: LPS during gestation, LPS-G+CJ: LPS during gestation + cherry juice, LPS-GL: LPS during gestation-lactation, LPS-GL+CJ: LPS during gestation-lactation + cherry juice.

The LPS-G and LPS-GL groups showed a significant increase in total leukocytes compared to the control group (F_4,25_ = 12.33, *p* < 0.0001). However, the LPS-G+CJ group was statistically equal to the control group (*p* < 0.05) (Figure 5A). As for total lymphocytes, only the LPS-G group showed a significant difference from the control group, but the administration of cherry juice decreased these values in the LPS-G+CJ group (F_4,25_ = 13.86, *p* < 0.0001) (Figure 5B). Total granulocytes also showed an increase for LPS-G group compared to the control group (F_4,25_ = 4.85, *p* = 0.0162) (Figure 5C).

### 2.3. Cell Counts of Hippocampal Regions

**Figure 6:**
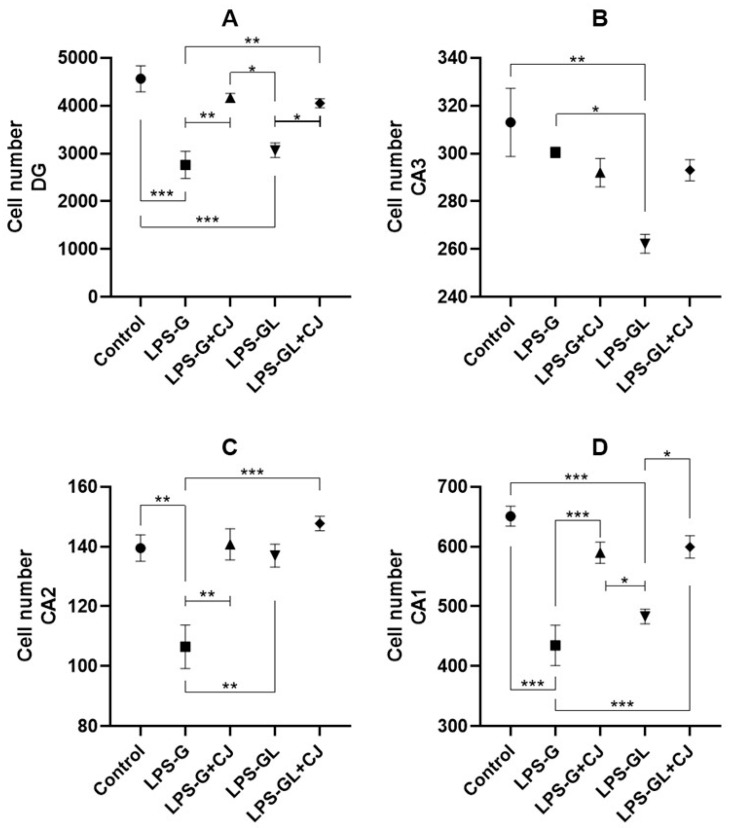
Mean ± SEM. Effects of cherry juice in maternal infection by lipopolysaccharide on hippocampal cell count. (**A**) Number of total cells in the dentate gyrus; (**B**) Number of total cells in the CA3; (**C**) Number of total cells in the CA2; (**D**) Number of total cells in the CA1. * *p* < 0.01, ** *p* < 0.001, *** *p* < 0.0001. (Tukey’s post hoc). n = 4. LPS-G: LPS during gestation, LPS-G+CJ: LPS during gestation + cherry juice, LPS-GL: LPS during gestation-lactation, LPS-GL+CJ: LPS during gestation-lactation + cherry juice.

Regarding the dentate gyrus region, the LPS-G and LPS-GL groups showed lower cell counts compared to the control group (F_4,15_ = 15.01, *p* < 0.0001). However, the cell counts in the dentate gyrus of the LPS-G+CJ and LPS-GL+CJ groups were statistically equal to the control group (*p* < 0.05) (Figure 6A). With respect to the CA3 region, only the LPS-GL group showed a lower cell count compared to the control group (F_4,15_ = 6.79, *p* = 0.0018) (Figure 6B). On the other hand, for the cell counts in the CA2 region, the LPS-G showed significant differences compared to the control group (F_4,15_ = 6.77, *p* = 0.0019); however, the LPS-G+CJ group was statistically equal compared to the control group (*p* < 0.05) (Figure 6C). A decrease in cell number in the CA1 region was observed in the LPS-G and LPS-GL groups compared to the control group (F_4,15_ = 17.93, *p* < 0.0001); however, the administration of cherry juice increased the of cell number in the CA1 region in the LPS-G+CJ and LPS-GL+CJ groups, which were statistically equal to the control group (*p* < 0.05) (Figure 6D).

## 3. Discussion

Several studies have shown that lipopolysaccharide can affect the brain and immune system during and after pregnancy through the inflammatory response, increasing blood-forming elements such as leukocytes [26,27,28,29]. In our study, we observed an increase in total leukocytes in the LPS-exposed groups. This increase was observed in lymphocyte and granulocyte counts, mainly in the LPS-G group. Kirsten et al. [30] mentioned that prenatal LPS exposure increased blood leukocyte levels and altered the immune response in adulthood. Similarly, Kasawara et al. [31] reported an increase in leukocytes, mainly in the number of lymphocytes, granulocytes, and monocytes in an in vivo model of lipopolysaccharide-induced inflammation, with changes in maternal and fetal morbidity. On the other hand, the percentage of lymphocytes is an important indicator of immune status, while neutrophil granulocytes and their percentages are closely related to inflammation and malnutrition [32]. Interestingly, in our study the cherry juice reduced leukocytes and lymphocytes in the offspring of the LPS-G-CJ group. It has been reported that polyphenols found in the leaves of *Quercus coccinea* can reduce the number of white blood cells and neutrophils [33].

On other hand, we observed hyperactivity-type behavior in the offspring of LPS-exposed groups during gestation (LPS-G) and gestation-lactation (LPS-GL) periods, with increased speed and total acceleration during the OF test, especially in the LPS-GL group. Similar data have been reported previously, with one study mentioned that prenatal administration of LPS caused locomotor hyperactivity in offspring at postnatal day 60 [34]. In addition, Romero et al. [35] showed that chronic exposure to LPS during pregnancy caused hyperactivity at postnatal day 56–57. We observed that LPS administration during gestation and gestation-lactation periods caused anxiogenic-like behavior and an increment in locomotor activity in the LPS-G and LPS-GL groups. This could be attributed to the fact that maternal infection by LPS during gestation and gestation-lactation periods decreased the number of cells in the regions of hippocampus, mainly in CA1 and dentate gyrus. This suggests an alteration in the process of embryonic neurogenesis in the offspring leading anxiogenic-like behavior and increased locomotor activity in the adult stage. However, the administration of cherry juice reduced the anxiogenic effect induced by the presence of LPS and inhibited the development of hyperactivity type behaviors.

In addition, systemic administration of LPS during gestation in murine models has been associated with behavioral abnormalities such as impaired memory and learning [36,37]. It could be associated with a reduction in the cell numbers in the regions of the hippocampus, particularly in CA1 and dentate gyrus. The dentate gyrus is an important region due to its involvement in episodic memory and neurogenesis. However, dietary compounds such as anthocyanins present in food can be used during gestation and lactation for prevention and treatment to improve memory of in the offspring. In our study, the administration of cherry juice provided a neuroprotective effect, improving short- and long-term memory in the offspring of the LPS-G+CJ and LPS-GL+CJ.

Tart cherry supplementation has been reported to improve working memory in aged rats [38]. In the present study, the administration of cherry juice improved the short- and long-term memory in the offspring of the LPS-G+CJ and LPS-GL+CJ groups according to NOR test. Several studies have reported that anthocyanins improve cognitive deficits in both animals and humans [39]. One study showed that consumption of anthocyanin-rich cherry juice improved memory in older adults [40]. It could be attributed to the neuroprotective effect of anthocyanins. A previous study showed that anthocyanins-rich blackberry juice administered to pregnant rats increased cell density in the dentate gyrus of the offspring. In the same way, blue corn tortillas administered during gestation rats increased the cell density in the dentate gyrus of the offspring [17,25]. Likewise, anthocyanins have been shown to increase synaptic plasticity, improving memory and learning after LPS infection in the cerebral cortex and hippocampus [41].

It has been reported that LPS administration disrupts with proper brain development and reduces the number of cells in the CA1 and CA3 regions of the hippocampus [42]. Furthermore, Vargas-Caraveo et al. [43], where they administered three different LPS strains in a murine model where the group exposed to the *E. coli* strain showed significant changes in the cell density compared to the control group. In our study, we observed a decrease in the number of cells in the CA1 and dentate gyrus of the hippocampus in the offspring of the LPS-G and LPS-GL groups; however, the administration of cherry juice showed a neuroprotective property due to the positive effect on the number of cells in the dentate gyrus and CA1 of the offspring of LPS-G+CJ and LPS-GL+CJ. In relation to our findings, the administration of cherry juice in the LPS-induced groups (LPS-G+CJ and LPS-GL+CJ) improved cognitive performance in the short-term and long-term memory compared to the LPS-exposed groups (LPS-G and LPS-GL), which related to the histological analysis, where the LPS-exposed groups showed a significant loss of cells in the CA1 and dentate gyrus regions of hippocampus compared to the control group, but the administration of cherry juice reduced the cell loss, demonstrating the neuroprotective effect of cherry juice during the ontogeny of the offspring.

Studies that follow offspring after birth may not be sufficient to detect late or chronic effects related to LPS exposure during pregnancy and after birth. Differences in the immune response between infected and uninfected mothers may have important implications for the health of the offspring. Because infection activates the maternal immune system, it can lead to an inflammatory response that affects both mother and offspring. Although uninfected mothers also contribute antibodies through breast milk, the type and amount may be different from those of mothers who have undergone an infectious process. Exposure to infection may enhance the transfer of antibodies and protective immune cells to the offspring, while uninfected mothers may provide a more stable environment but with different immune profiles.

## 4. Materials and Methods

### 4.1. Preparation of Cherry Juice

The cherries (*Prunus avium*) were purchased from a local fruit market (Unifrutti trades SpA, Santiago, Chile) at a commercial stage of ripeness, harvested in autumn in the VII Teno Linares region of Chile. The cherries were washed with a 0.1% sodium hypochlorite solution to remove surface contaminants. The cherry seed was removed, and mash cherry was obtained using a homogenizer and it was placed on Petri dish in a microwave oven (Panasonic (Kadoma, Osaka, Japan), 2450 MHz) [24]. The microwave energy applied was 0.54 KJ/g of cherry mash [44]. The content of total anthocyanins was evaluated according to the pH differential method [45]. The juice was diluted with buffer potassium chloride 0.025 M (pH 1) and buffer sodium acetate 0.4 M (pH 4.5). Then, the absorbance was measured at 515 and 700 nm with a spectrophotometer (Genesys 10S UV/VIS, Thermo Scientific (Waltham, MA, USA)), and the data were expressed as mg of cyanidin-3-glucoside equivalent/L (C3GE)/L. The total anthocyanin content was 329 mg C3GE/L. The nutritional composition of the cherry juice was also analyzed. The protein level of the cherry juice was 0.1 g/100 g of sample, while the fat, ash, and carbohydrate content were 1.0, 2.6. and 8.0 g/100 g of sample, respectively (wet weight basis). A total of 100 g of cherry juice provides 41.4 kcal.

### 4.2. Animal Study

This study was conducted under the approval of the ethical committee for the use and care of laboratory animals (CICIAL-ICS: 2020-02/002) and the research registration and evaluation system 27928202339 of the Universidad Veracruzana, and according to the Official Mexican Standard, NOM-062-ZOO-1999 [46]. Animals were obtained from the Institute of Neuroethology and were housed under a 12 h light/dark cycle with ad libitum access to food and water.

Female rats around 250 g were mated during the estrus phase of the estrous cycle, with mating considered as embryonic day (E) 0. A total of 30 pups were evaluated. The groups had n = 6 and were divided as follows: control (administered with saline solution); LPS-G (lipopolysaccharide administration during gestation); LPS-G+CJ (lipopolysaccharide administration during gestation + cherry juice); LPS-GL (lipopolysaccharide administration during gestation-lactation); LPS-GL+CJ (lipopolysaccharide administration during gestation-lactation + cherry juice).

Administration of saline solution (0.9% NaCl) for the control group and LPS bacterial solution (*E. coli*; serotype O111, Sigma-Aldich, Darmstadt, Germany) at a dose of 50 µg/kg [47] for the LPS-G, LPS-G+CJ, LPS-GL, and LPS-GL+CJ groups was performed by intraperitoneal injections into the pregnant dams on days E10, E12, and E14. The day of birth was considered as P0. Offspring of the LPS-GL and LPS-GL+CJ groups received a subcutaneous injection of the same LPS dose at P7. The cherry juice was administered intragastric to pregnant dams during gestation and gestation-lactation periods, daily using 2 mL of the cherry juice. This dosage provided 0.65 mg of anthocyanins per 0.25 kg of body weight. The human dose equivalent based on body surface area method was 0.49 mg/kg [47,48,49]. Offspring were weaned at postnatal day 21 (P21) and once the study age (P90) was reached, behavioral tests were performed, followed by euthanasia for blood cell analysis and histological study of the hippocampus.

### 4.3. Behavioral Studies

The behavioral tests were conducted P90 of male offspring including the open field (OF), novel object recognition (NOR), and elevated plus maze (EPM) tests, ensuring controlled conditions for all animals. The software idTracker 2.1 [50] and MOTUS 1.5.4 [51] were used to analyze the recorded videos.

The OF test allowed us to evaluate the exploratory activity and locomotor activity of the rats. For this purpose, each rat was placed in the central part of the arena (90 × 45 cm) for a period of 5 min. Parameters of speed, acceleration, and total distance were quantified. The NOR test was used to evaluate episodic short-term memory (STM) and long-term memory (LTM). For this, the animals were placed in the central part of the arena (90 × 90 × 45 cm). The test consisted of four phases: habituation, training, STM, and LTM, each lasting 5 min. The number of interactions and the time spent with both objects were evaluated, as well as the parameters of speed, acceleration, and total distance.

For the EPM test, the number of entries into the open and closed arms and the time spent in the open and closed arms were evaluated. This consists of two opposite open arms (50 × 10 cm), two opposite closed arms painted black (50 × 10 cm) with 40 cm walls, and a central part (10 × 10 cm). The arena was placed 50 cm above the ground and the test had a duration of 5 min. Once the tests were completed, blood samples were collected for white blood cell analysis, as well as the brain for hippocampal cell counts.

### 4.4. Leukocyte Analysis

A blood count of leukocytes, lymphocytes and granulocytes was performed. Blood samples were collected 24 h after the behavioral tests. The pups were previously anesthetized, and once unconscious, the samples were collected intracardially and stored in EDTA K2 Vacutainer tubes (Thermo Scientific (Waltham, MA, USA)). The analysis was carried out using the AbaxysTM VetScanTM instrument (Zoetis (Parsippany, NJ, USA)).

### 4.5. Hippocampal Cell Count

The number of cells in the hippocampus regions CA1, CA2, and CA3 and the dentate gyrus were analyzed. Rat brains were obtained by transcardial perfusion followed by dehydration in different concentrations of ethanol. After fixation, the brains were embedded in paraffin and sections in 10 µm thick slices using a microtome (Reichert-Jung 820 II, Buffalo, NY, USA) and stained with Nissl’s stain. Microphotographs were taken with a visible light microscope (Leica model DM750, Wetzlar, Germany) equipped with a camera (Infinity 1-5C Lumera, Ottawa, ON, Canada) using INFINITY CAPTURE software (version 6.5.2). The hippocampal regions were analyzed between the level of coronal plate 61 was used (interaural 5.64 mm and Bregman −3.36 mm) using the atlas “The rat brain in stereotaxic coordinates” [52]. ImageJ (version 1.51 h) was used to count the hippocampal regions.

### 4.6. Statistical Analysis

Datasets were presented as mean ± standard error of the mean (SEM). For the OF test, leukocyte analysis and cell counts of hippocampal regions were used, and one-way analysis of variance (ANOVA) was used when the data followed a normal distribution and equal variance. For the NOR test, familiar objects and novel objects were used as factors, and for the EPM test, open arms and closed arms were used as factors, and two-way ANOVA was used. Statistical significance was set at *p* < 0.05 followed by Tukey’s post hoc test. GraphPad Prism statistical software version 8.0 (GraphPad Software, Inc., Boston, MA, USA, 2016) was used for all analyses.

## 5. Conclusions

Maternal infection by LPS during gestation and gestation-lactation periods altered the leukocyte and lymphocyte content in the blood of the pups, causing alterations in different regions of the hippocampus, mainly in CA1 and dentate gyrus as well as decrease in short- and long-term memory in the adult stage, demonstrating the chronic effect of LPS on the ontogeny of the pups. However, the administration of cherry juice provided a positive effect on the leukocyte and lymphocyte content in the pups, as well as on the cell count in the dentate gyrus and CA1 regions improving short- and long-term memory. In addition, the cherry juice administration reduced the anxiogenic effect caused by LPS and inhibited the increment of locomotor activity. The administration of cherry juice during maternal LPS-induced infection attenuated cognitive impairments in the offspring, supporting its potential as a neuroprotective dietary intervention. Nevertheless, further studies are warranted, including animal experiments involving females to explore sex-specific neuroprotective mechanisms, as well as clinical trials to assess its efficacy and safety in humans.

## Data Availability

The datasets presented in this article are not readily available because the data are part of an ongoing study and to prevent commercial use of this research. Requests to access the datasets should be directed to the corresponding author.
